# Accessory cardiac bronchus causing recurrent pulmonary infection

**DOI:** 10.1590/S1806-37132014000400014

**Published:** 2014

**Authors:** Gláucia Zanetti, Bruno Hochhegger, Marcos Duarte Guimarães, Edson Marchiori

**Affiliations:** Graduate Program in Radiology, Federal University of Rio de Janeiro, Rio de Janeiro; and Professor of Clinical Medicine, Petrópolis School of Medicine, Petrópolis, Brazil; Santa Casa Hospital Complex in Porto Alegre; and Professor of Radiology, Federal University of Health Sciences of Porto Alegre, Porto Alegre, Brazil; A.C. Camargo Cancer Center, São Paulo, Brazil; Federal University of Rio de Janeiro, Rio de Janeiro, Brazil

## To the Editor:

**Figure 1:**
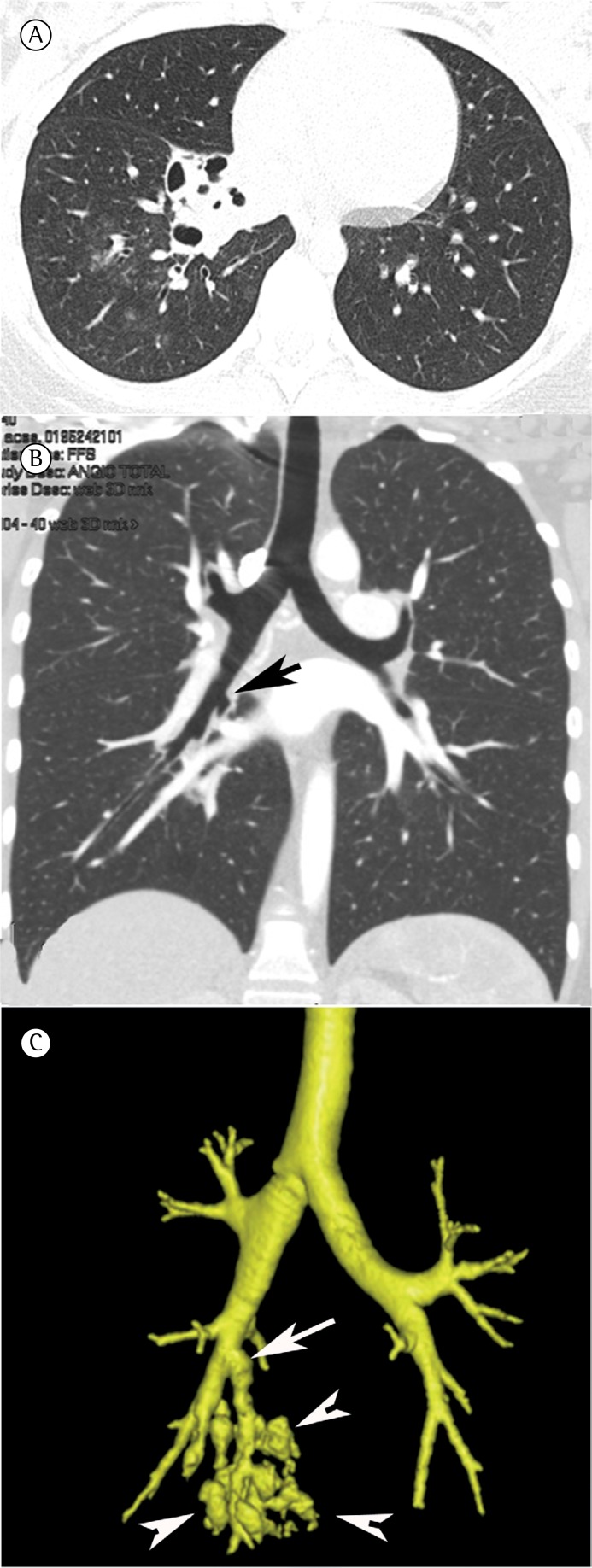
In A, an axial CT image demonstrating consolidations with cystic areas in the right paracardiac region. In B, a reformatted coronal image showing an accessory cardiac bronchus (arrow) arising from the medial wall of the intermediate bronchus. In C, threedimensional shaded surface display coronal reformatting, showing the accessory cardiac bronchus (arrow) and a correspondent lobule with cystic dilatations (arrowheads).

A 15-year-old female patient presented to our hospital with a history of recurrent pneumonia and complaints of productive cough and episodes of bronchospasm. Physical examination revealed crackles in the right hemithorax. Laboratory test findings were normal. A chest X-ray showed right paracardiac opacities. Axial CT (Figure 1A) demonstrated consolidations with cystic areas in the right paracardiac region. A reformatted coronal image showed an accessory cardiac bronchus (ACB; Figure 1B, arrow) arising from the medial wall of the intermediate bronchus. Three-dimensional shaded surface display coronal reformatting showed the ACB (Figure 1C, arrow) and a correspondent lobule with cystic dilatations (arrowheads). Bronchoscopy confirmed the presence of the ACB arising from the intermediary bronchus. Bronchoalveolar lavage and cultures were negative for Mycobacterium spp. and fungi. Surgery demonstrated infected cystic structures and small bronchioles and alveoli with retained secretions distally to the ACB.

Bronchial division anomalies are common, although most are encountered incidentally in asymptomatic adults. They might be isolated or associated with a variety of other congenital disorders.^(^ 1 ^)^ ACB is a rare congenital anomaly of the tracheobronchial tree, characterized by an anomalous bronchus originating from the intermediate bronchus opposite to the origin of the right upper lobe bronchus or originating from the medial wall of the right main bronchus. ^(1-3) ^From its origin, it runs medially and caudally toward the heart.^(2) ^An ACB might be a short, blind-ended structure or a long, branching bronchus that develops into a series of small bronchioles, which might end in vestigial parenchymal tissue in the bronchioles or in cystic degeneration, or it might be associated with small amounts of pulmonary parenchyma.^(^ 1 ^,^ 3 ^)^

Most patients with ACB are asymptomatic, and the anomaly is discovered incidentally during bronchoscopy or imaging studies conducted for unrelated reasons.^(1,4) ^However, an ACB can become symptomatic due to recurrent infection, empyema, hemoptysis, and malignant transformation.^(1,2,4,5) ^These symptoms are caused by the accumulation of secretions in the ACB, leading to inflammation and infection, extensive microvascularization, and hemoptysis, especially when the ACB is long or has an accessory lobe.^(2,4) ^Thus, the short type of ACB tends to be asymptomatic, whereas the accessory-lobed and long diverticular types are more susceptible to complications.^(^ 5 ^)^

Histological examination suggested that the specimen resected from our patient was the accessory bronchus, including an accessory lobe with retained secretions. The finding of scar tissue, but no alveoli, on the peripheral accessory lobe suggested that it had been deteriorated or ruptured by constant infection, leading to bronchopneumonia and empyema.^(^ 4 ^)^

An ACB is not generally visible on chest X-ray, but it can be visualized well with other imaging modalities. Surgical resection of a long ACB or of one with an accessory lobe is advised as soon as symptoms occur.^(^ 4 ^,^ 5 ^)^

In conclusion, pulmonologists and radiologists should recognize normal bronchial anatomy as well as developmental bronchial anomalies because this is important to establish a correct diagnosis. Although an ACB is not pathological per se, it is occasionally associated with clinical symptoms and complications. 
