# Supplementary material for: Accessory cardiac bronchus causing recurrent pulmonary infection
Source: J Bras Pneumol. 2014 Jul-Aug;40(4):448–9. doi: 10.1590/S1806-37132014000400014 (PMC4201177; doi:10.1590/S1806-37132014000400014)
Supplement: Translation

# Translation: Brônquio cardíaco acessório causando infecções respiratórias de repetição

**Authors:** Gláucia Zanetti, Bruno Hochhegger, Marcos Duarte Guimarães, Edson Marchiori

**Affiliations:** Programa de Pós-Graduação em Radiologia, Universidade Federal do Rio de Janeiro, Rio de Janeiro (RJ); e Professora de Clínica Médica, Faculdade de Medicina de Petrópolis, Petrópolis (RJ) Brasil; Complexo Hospitalar Santa Casa de Misericórdia de Porto Alegre; e Professor de Radiologia, Universidade Federal de Ciências da Saúde de Porto Alegre, Porto Alegre (RS) Brasil; A.C. Camargo Cancer Center, São Paulo (SP) Brasil; Universidade Federal do Rio de Janeiro, Rio de Janeiro (RJ) Brasil

## Ao Editor:

Uma paciente de 15 anos de idade apresentou-se em nosso hospital com história de pneumonia recorrente e queixas de tosse produtiva e episódios de broncoespasmo. O exame físico revelou estertores crepitantes no hemitórax direito. Os achados laboratoriais foram normais. A radiografia de tórax mostrou opacidades paracardíacas direitas. A TC axial (Figura 1A) mostrou consolidações com áreas císticas na região paracardíaca direita. Uma imagem coronal reformatada mostrou um brônquio cardíaco acessório (BCA; Figura 1B, seta) proveniente da parede medial do brônquio intermediário. A reformatação coronal tridimensional por exibição de superfície sombreada mostrou o BCA (Figura 1C, seta) e um lóbulo correspondente com dilatações císticas (pontas de seta). A broncoscopia confirmou a presença de BCA proveniente do brônquio intermediário. A lavagem broncoalveolar e as culturas foram negativas para *Mycobacterium* spp. e fungos. A cirurgia revelou estruturas císticas infectadas e pequenos bronquíolos e alvéolos com secreções retidas, distais ao BCA. 

**Figura 1:** Em A, imagem de TC axial mostrando consolidações com áreas císticas na região paracardíaca direita. Em B, imagem coronal reformatada mostrando um brônquio cardíaco acessório (seta) proveniente da parede medial do brônquio intermediário. Em C, reformatação coronal tridimensional por exibição de superfície sombreada, mostrando o brônquio cardíaco acessório (seta) e um lóbulo correspondente com dilatações císticas (pontas de seta).

Anomalias de divisões brônquicas são comuns, embora a maioria seja encontrada por acaso em adultos assintomáticos. Podem estar isoladas ou acompanhadas de várias outras doenças congênitas.^(^ 1 ^)^ Uma rara anomalia congênita da árvore traqueobrônquica, o BCA é um brônquio anômalo proveniente do brônquio intermediário oposto à origem do brônquio do lobo superior direito ou proveniente da parede medial do brônquio principal direito.^(^ 1 ^-^ 3 ^)^ A partir de sua origem, estende-se medial e caudalmente até o coração.^(^ 2 ^)^ Um BCA pode ser uma estrutura curta e sem abertura ou um brônquio longo ramificado em uma série de pequenos bronquíolos, que pode terminar em tecido parenquimatoso residual nos bronquíolos ou em degeneração cística, ou pode estar associado a pequenas quantidades de parênquima pulmonar.^(^ 1 ^,^ 3 ^)^

A maioria dos pacientes com BCA é assintomática, e a anomalia é descoberta por acaso durante uma broncoscopia ou exames de imagem realizados por outros motivos, sem relação com a anomalia.^(^ 1 ^,^ 4 ^)^ No entanto, um BCA pode se tornar sintomático em virtude de infecção recorrente, empiema, hemoptise e transformação maligna.^(^ 1 ^,^ 2 ^,^ 4 ^,^ 5 ^)^ Esses sintomas são causados pela acumulação de secreções no BCA e levam a inflamação e infecção, microvascularização extensa e hemoptise, especialmente quando o BCA é longo ou tem um lobo acessório.^(^ 2 ^,^ 4 ^)^ Assim, os BCA curtos tendem a ser assintomáticos, ao passo que os tipos lobo acessório e longo apendicular são mais propensos a complicações.^(^ 5 ^)^

O exame histológico sugeriu que o espécime ressecado de nossa paciente era o brônquio acessório, incluindo um lobo acessório com secreções retidas. O achado de tecido cicatricial, porém sem alvéolos, no lobo acessório periférico sugeriu que havia se deteriorado ou rompido em virtude de infecção constante, levando a broncopneumonia e empiema.^(^ 4 ^)^

Um BCA geralmente não é visível na radiografia de tórax, mas pode ser visto nitidamente por meio de outros métodos de imagem. Recomenda-se a resseção cirúrgica de um BCA longo ou de um com lobo acessório assim que ocorram sintomas.^(^ 4 ^,^ 5 ^)^

Conclui-se que pneumologistas e radiologistas devem reconhecer a anatomia brônquica normal e anomalias do desenvolvimento brônquico, pois isso é importante para estabelecer o diagnóstico correto. Embora um BCA não seja patológico em si, associa-se às vezes a sintomas clínicos e complicações.
